# Supplementary material for: Decreasing Species Richness with Increase in Elevation and Positive Rapoport Effects of Crambidae (Lepidoptera) on Mount Taibai
Source: Insects. 2022 Dec 5;13(12):1125. doi: 10.3390/insects13121125 (PMC9783943; doi:10.3390/insects13121125)
Supplement: Supplementary file 1 [file insects-13-01125-s001.zip › Table S4.docx]

**Table S4**. Elevational distribution of sampled Specimens of Pyraustinae and Spilomelinae from Mount Taibai

| Subfamily | Scientific name | No. of specimens | Upper limit of altitude (m) | Mid-point of altitude (m) | Species range (m) |
| --- | --- | --- | --- | --- | --- |
| Pyraustinae | *Aglaops aurantialis* (Munroe & Mutuura, 1968) | 1 | 1136 | 1136 | - |
|  | *Anania egentalis* (Christoph, 1881) | 6 | 1972 | 1612 | 720 |
|  | *Anania verbascalis* (Denis & Schiffermüller, 1775) | 17 | 1972 | 1459.5 | 1025 |
|  | *Circobotys plebeia* (Munroe & Mutuura, 1969) | 5 | 2280 | 2029 | 502 |
|  | *Crypsiptya coclesalis* (Walker, 1859) | 1 | 2280 | 2280 | - |
|  | *Loxostege turbidalis* (Treitschke, 1829) | 1 | 1638 | 1638 | - |
|  | *Opsibotys fuscalis* (Denis & Schiffermüller, 1775) | 11 | 1880 | 1762 | 236 |
|  | *Ostrinia furnacalis* (Guenée, 1854) | 8 | 1972 | 1459.5 | 1025 |
|  | *Ostrinia scapulalis* (Walker, 1859) | 2 | 1088 | 1038.5 | 100 |
|  | *Proteurrhypara cuspidata* (Zhang, Li & Wang, 2002) | 6 | 1432 | 1388.5 | 100 |
|  | *Pseudebulea fentoni* (Butler, 1881) | 10 | 1432 | 1189.5 | 485 |
|  | *Pyrausta noctualis* (Yamanaka, 1978) | 40 | 1980 | 1463.5 | 1033 |
|  | *Sinibotys evenoralis* (Walker, 1859) | 1 | 1430 | 1430 | - |
|  | *Sitochroa palealis* (Denis & Schiffermüller, 1775) | 3 | 1778 | 1778 | 100 |
|  | *Sitochroa verticalis* (Linnaeus, 1758) | 1 | 1778 | 1778 | - |
|  | Pyraustinae sp. Meyrick, 1890 | 1 | 1136 | 1136 | - |
| Spilomelinae | *Agrotera nemoralis* (Scopoli, 1763) | 2 | 1252 | 1252 | 100 |
|  | *Botyodes diniasalis* (Walker, 1859) | 3 | 1972 | 1701 | 542 |
|  | *Bradina atopalis* (Walker, 1859) | 7 | 1252 | 1099.5 | 305 |
|  | *Camptomastix hisbonalis* (Walker, 1859) | 37 | 1972 | 1459.5 | 1025 |
|  | *Conogethes punctiferalis* (Guenée, 1854) | 2 | 947 | 913.5 | 100 |
|  | *Cotachena alysoni* (Whalley, 1961) | 9 | 1850 | 1398.5 | 903 |
|  | *Cotachena nepalensis* (Yamanaka, 2000) | 15 | 1542 | 1244.5 | 595 |
|  | *Diaphania perspectalis* (Walker, 1859) | 3 | 1432 | 1156 | 552 |
|  | *Eurrhyparodes bracteolalis* (Zeller, 1852) | 1 | 947 | 947 | - |
|  | *Glyphodes formosanus* (Shibuya, 1928) | 14 | 1980 | 1534 | 892 |
|  | *Glyphodes quadrimaculalis* (Bremer & Grey, 1853) | 21 | 1972 | 1426 | 1092 |
|  | *Goniorhynchus exemplaris* (Hampson, 1898) | 69 | 2080 | 1513.5 | 1133 |
|  | *Goniorhynchus marginalis* (Warren, 1896) | 129 | 2280 | 1613.5 | 1333 |
|  | *Haritalodes basipunctalis* (Fabricius, 1775) | 31 | 2080 | 1513.5 | 1133 |
|  | *Haritalodes derogate* (Fabricius, 1775) | 4 | 947 | 947 | - |
|  | *Herpetogramma luctuosalis* (Guenée, 1854) | 38 | 1778 | 1362.5 | 831 |
|  | *Herpetogramma magna* (Butler, 1879) | 9 | 1847 | 1397 | 900 |
|  | *Herpetogramma pseudomagna* (Yamanaka, 1976) | 9 | 1972 | 1511.5 | 921 |
|  | *Lamprosema commixta* (Butler, 1879) | 27 | 1644 | 1295.5 | 697 |
|  | *Lamprosema sibirialis* (Milliére, 1879) | 1 | 1549 | 1549 | - |
|  | *Maruca testulalis* (Fabricius, 1787) | 7 | 1972 | 1459.5 | 1025 |
|  | *Mecyna dissipatalis* (Lederer, 1863) | 17 | 1972 | 1459.5 | 1025 |
|  | *Mecyna flavalis* (Denis & Schiffermüller, 1775) | 1 | 1850 | 1850 | - |
|  | *Mecyna gracilis* (Butler, 1879) | 2 | 947 | 947 | 100 |
|  | *Mecyna tricolor* (Butler, 1879) | 7 | 1290 | 1118.5 | 343 |
|  | *Nacoleia charesalis* (Walker, 1859) | 5 | 1252 | 1099.5 | 305 |
|  | *Nacoleia maculalis* (Millière, 1879) | 5 | 1644 | 1295.5 | 697 |
|  | *Nagiella occultalis* (Ullah, Yang & Qiao, 2017) | 13 | 1432 | 1189.5 | 485 |
|  | *Nomophila nocteulla* (Denis & Schiffermüller, 1775) | 1 | 947 | 947 | - |
|  | *Nosophora* sp. Lederer, 1863 | 2 | 1182 | 1182 | 100 |
|  | *Omiodes poeonalis* (Walker, 1859) | 1 | 1051 | 1051 | - |
|  | *Omiodes tristrialis* (Bremer, 1864) | 2 | 1963 | 1906.5 | 113 |
|  | *Palpita nigropunctalis* (Bremer, 1864) | 34 | 2280 | 1613.5 | 1333 |
|  | *Nagiella quadrimaculalis* (Hampson, 1898) | 16 | 1850 | 1450.5 | 799 |
|  | *Pleuroptya balteata* (Fabricius, 1798) | 1 | 1136 | 1136 | 100 |
|  | *Pleuroptya chlorophanta* (Butler, 1878) | 32 | 1847 | 1397 | 900 |
|  | *Pleuroptya expictalis* (Christoph, 1881) | 1 | 1432 | 1432 | - |
|  | *Pleuroptya inferior* (Hampson, 1898) | 6 | 1972 | 1459.5 | 1025 |
|  | *Pleuroptya mundalis* (South, 1901) | 12 | 1432 | 1189.5 | 485 |
|  | *Pleuroptya sabinusalis* (Walker, 1859) | 1 | 1290 | 1290 | - |
|  | *Pycnarmon cribrate* (Fabricius, 1794) | 48 | 2280 | 1613.5 | 1333 |
|  | *Pycnarmon pantherata* (Butler, 1878) | 9 | 1880 | 1508 | 744 |
|  | *Rehimena phrynealis* (Walker, 1859) | 39 | 1850 | 1398.5 | 903 |
|  | *Spoladea recurvalis* (Fabricius, 1775) | 3 | 947 | 913.5 | 67 |
|  | *Syllepte invalidalis* (South, 1901) | 66 | 1972 | 1459.5 | 1025 |
|  | *Syllepte segnalis* (Leech, 1889) | 19 | 1972 | 1577 | 790 |
|  | *Syllepte taiwannalis* (Shibuya, 1928) | 4 | 1432 | 1342 | 180 |
|  | *Tabidia strigiferalis* (Hampson, 1900) | 2 | 1432 | 1342 | 180 |
|  | *Tylostega pectinate* (Du & Li, 2008) | 53 | 1880 | 1525 | 710 |
|  | *Tylostega serrata* (Du & Li, 2008) | 36 | 2280 | 1634.5 | 1291 |
|  | *Tylostega tylostegalis* (Hampson, 1900) | 35 | 1850 | 1445.5 | 809 |
|  | *Tyspanodes hypsalis* (Warren, 1891) | 44 | 1972 | 1480.5 | 983 |
|  | *Tyspanodes striata* (Butler, 1879) | 44 | 2280 | 1766 | 1028 |
|  | *Udea costalis* (Eversmann, 1852) | 21 | 2280 | 2180 | 200 |
|  | *Udea ferrugalis* (Hübner, 1796) | 2 | 1638 | 1534 | 208 |
|  | *Udea lugubralis* (Leech, 1889) | 5 | 1963 | 1549.5 | 827 |
|  | *Udea* sp. Guenée, [1845]1844 | 2 | 1963 | 1963 | 100 |
