# Supplementary figures and images for: Decreasing Species Richness with Increase in Elevation and Positive Rapoport Effects of Crambidae (Lepidoptera) on Mount Taibai
Source: Insects. 2022 Dec 5;13(12):1125. doi: 10.3390/insects13121125 (PMC9783943; doi:10.3390/insects13121125)

Figure S1. The NJ tree is based on COI gene sequences.

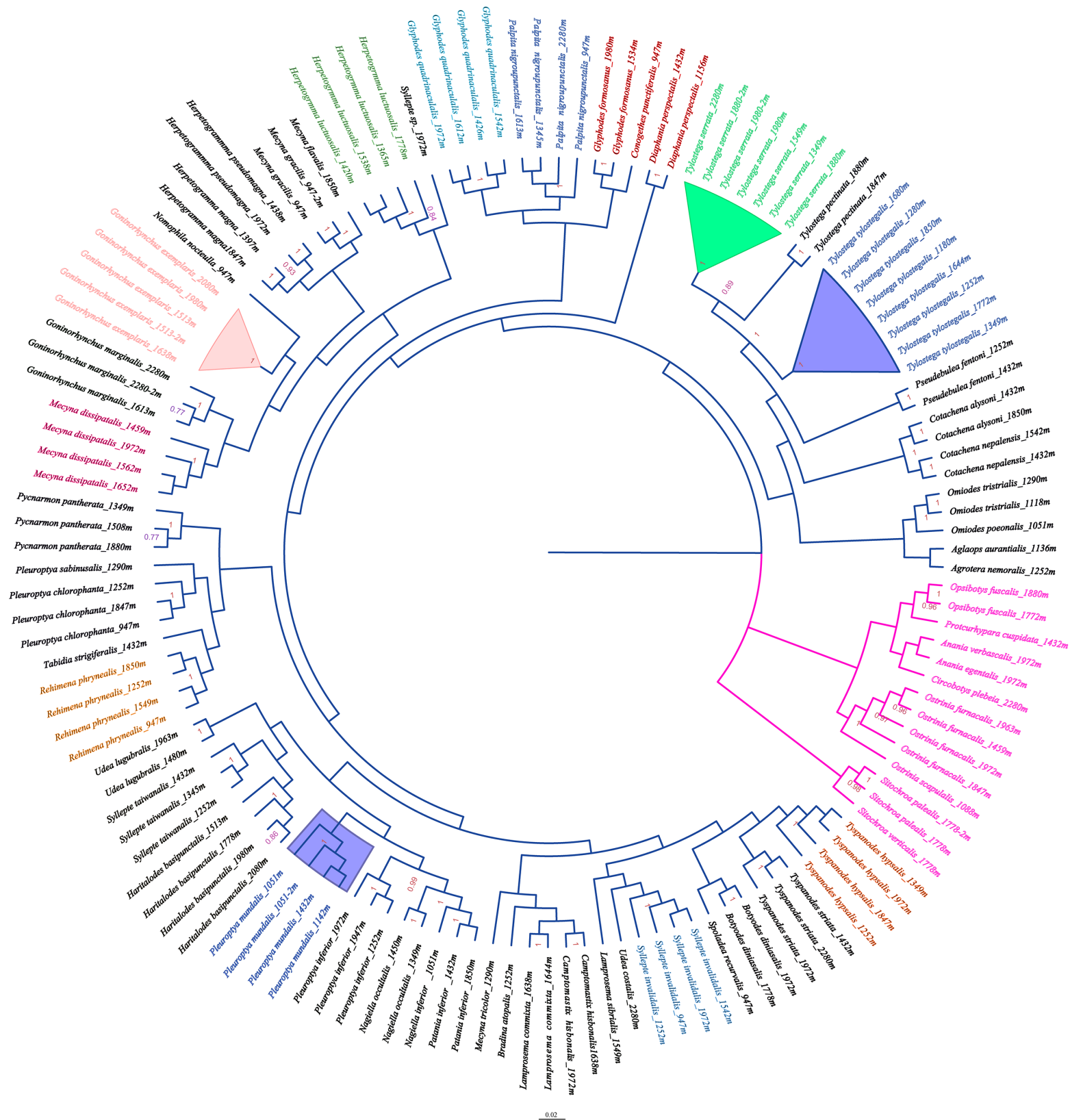

Supplement: Supplementary file 1 [file insects-13-01125-s001.zip › Figure S1.pdf]
